# Risk Factors of Undiagnosed Diabetes Mellitus among Korean Adults: A National Cross-Sectional Study Using the KNHANES Data

**DOI:** 10.3390/ijerph18031195

**Published:** 2021-01-29

**Authors:** Sangwon Lee, Kwang Sun Ryu, Ha Ye Jin Kang, Na Young You, Kui Son Choi, Yul Hwangbo, Jae Wook Lee, Hyo Soung Cha

**Affiliations:** 1 Cancer Data Center, National Cancer Control Institute, National Cancer Center, Gyeonggi-do, Goyang-si 10408, Korea; clstrange31@gmail.com (S.L.); niceplay13@ncc.re.kr (K.S.R.); khyj0302@ncc.re.kr (H.Y.J.K.); nayoung0715@ncc.re.kr (N.Y.Y.); kschoi@ncc.re.kr (K.S.C.); 2 Graduate School of Cancer Science and Policy, National Cancer Center, Gyeonggi-do, Goyang-si 10408, Korea; 3 Healthcare AI Team, Healthcare Platform Center, National Cancer Center, Gyeonggi-do, Goyang-si 10408, Korea; yulhwangbo@ncc.re.kr; 4 Division of Endocrinology, Department of Internal Medicine, National Cancer Center, Gyeonggi-do, Goyang-si 10408, Korea; 5 Division of Nephrology, Department of Internal Medicine, National Cancer Center, Gyeonggi-do, Goyang-si 10408, Korea; jwleemd@ncc.re.kr

**Keywords:** undiagnosed diabetes mellitus, access to healthcare, health behaviors, diabetes mellitus prevention, national survey analysis

## Abstract

In this cross-sectional study, we investigated the baseline risk factors of diabetes mellitus (DM) in patients with undiagnosed DM (UDM). We utilized the Korean National Health and Nutrition Examination Survey (KNHANES) 2010–2017 data. Data regarding the participants’ demographic characteristics, health status, health determinants, healthcare accessibility, and laboratory tests were gathered to explore the differences between the DM, UDM, and without-DM groups. Among the 64,759 individuals who participated in the KNHANES 2010–2017, 32,611 individuals aged ≥20 years with fasting plasma glucose levels of <100 or ≥126 mg/dL were selected. The odds ratios (ORs) regarding family history of diabetes and the performance of national health and cancer screening tests were lower in the UDM group than in the DM group (adjusted OR: 0.54; 95% confidence interval (CI): 0.43, 0.66; adjusted OR: 0.74; 95% CI: 0.62, 0.89; adjusted OR: 0.71; 95% CI: 0.60, 0.85). The ORs of hypertension and obesity were higher in the UDM group than in the DM group (adjusted OR: 1.32; 95% CI: 1.06, 1.64; adjusted OR: 1.80; 95% CI: 1.37, 2.36, respectively). Patients with UDM were more likely to be exposed to DM-related risk factors than those with and without DM. Public health interventions to prevent UDM development are necessary.

## 1. Introduction

Diabetes mellitus (DM), a group of metabolic disorders characterized by high blood glucose levels, is a major global health issue. Approximately 451 million people worldwide were estimated to have diabetes in 2017, and this value is expected to increase to 693 million by 2045 [1]. Additionally, the global economic burden of DM has been estimated to substantially increase by 2030 [2]. In Korea, the estimated age-standardized prevalence rates of diabetes for men and women during 2013–2015 were 12.9% and 9.3%, respectively, showing significant increases from the values observed in 2005 [3]. However, not only has the prevalence increased, but also, approximately half of all diabetes cases, both worldwide and in Korea, remain undiagnosed [1,3,4].

Understanding undiagnosed DM (UDM) is critical to mitigating the substantial growth of the DM burden. People with DM are at a high risk of developing several life-threatening and severe complications, such as coronary artery disease, peripheral arterial disease, stroke, neuropathy, nephropathy, and retinopathy [5,6]. As DM is asymptomatic, people with UDM may maintain their undiagnosed status without receiving any treatment and are subsequently at a higher risk of developing severe DM-related complications. Thus, it is essential to understand the characteristics of people with UDM to build a strategy for public health intervention against the growing population of those with DM. However, the characteristics of Koreans with UDM have not been established, especially compared to those of people with DM.

Therefore, this study aimed to investigate the characteristics of people with UDM in terms of demographic characteristics, health status, health determinants, healthcare accessibility, and laboratory testing. The results of this study would aid in the formulation of public health messages targeted at people with UDM for early detection and lifestyle intervention.

## 2. Materials and Methods

The present study included data from the Korean National Health and Nutrition Examination Survey (KNHANES) V, VI, and VII, which were conducted from 2010 to 2017 by the Korean Ministry of Health and Welfare, on the basis of a stratified and multistage probability cluster sampling design. The KNHANES has a cross-sectional design and is based on a non-duplicate complex sample, comprising nationally representative civilians [7]. People who participated in the survey signed an informed consent form. In addition, the KNHANES was approved by the Institutional Review Board (IRB) of the Korean Centers for Disease Control and Prevention (KCDC) [IRB: 2010-02CON-21-C; 2011-02CON-06-C; 2012-01EXP-01-2C; 2013-07CON-03-4C; 2013-12EXP-03-5C; 2018-01-03-P-A]. All methods were conducted in accordance with relevant guidelines and regulations. Approval from the IRB to perform this study was not required for the following reasons: (1) The KNHANES data do not include personal information, and each individual was assigned a unique personal identification number. (2) The KNHANES is open to the public and can be accessed via the following link: https://knhanes.cdc.go.kr.

**Figure 1:**
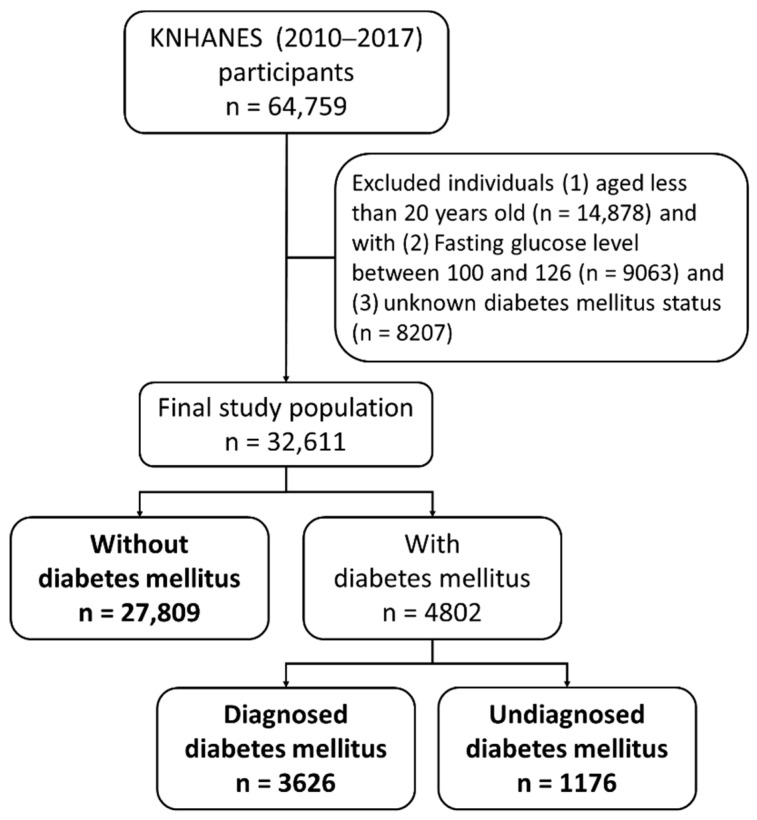
Flowchart presenting the selection of the individuals in the without-diabetes-mellitus (without-DM), with-DM, and undiagnosed-DM (UDM) groups; KNHANES, Korean National Health and Nutrition Examination Survey.

The following participants were excluded in the selection of the study population: (1) Korean adults aged <20 years (*n* = 14,878); (2) people whose fasting plasma glucose (FPG) levels were ≥100 mg/dL and <126 mg/dL (*n* = 9063); and (3) people with unknown DM status (*n* = 8207) (Figure 1). Those with FPG levels of ≥100 mg/dL and <126 mg/dL have been defined as having prediabetes status [8]; thus, they could not be assigned to either group (with or without DM group, or the UDM group). People who had not fasted for at least 8 h and whose values for the health interview survey regarding their diabetes status were missing were assigned to the unknown DM group [9]. The participants were classified into without-DM, DM, and UDM groups. DM was defined following the recommendations by the KCDC as follows: (1) FPG level of ≥126 mg/dL, (2) diabetes diagnosis by a medical doctor, or (3) the use of oral hypoglycemic agents or insulin injection. The UDM group comprised people whose FPG values were ≥126 mg/dL, but who answered on the questionnaire that they had never been diagnosed with DM [10]. The group without DM showed the following characteristics: (1) FPG levels of <100 mg/dL, (2) no history of diabetes diagnosis by a medical doctor, and (3) no use of oral hypoglycemic agents or insulin injection.

We selected 31 variables for which the relevant questionnaire and answers had not changed from 2010 to 2017. The definition of the selected variables was determined according to the KNHANES guidelines (https://knhanes.cdc.go.kr/knhanes/). Data on demographic characteristics, such as age, sex (male/female), educational attainment (high school or less/more than high school), marital status (yes/no), and income level (low/middle/high), were collected. We selected the health-status-related variables that could affect DM status, such as family history of diabetes (yes/no), hypertension status (normal/at high risk/hypertension), obesity (underweight/normal weight/overweight/obese) levels, and body mass index (BMI) (kg/m^2^). Obesity variables were classified based on the following BMI categories: underweight, BMI < 18.5 kg/m^2^; normal weight, 18.5 kg/m^2^ ≤ BMI < 25 kg/m^2^; overweight, 25 kg/m^2^ ≤ BMI < 30 kg/m^2^; and obese, BMI ≥ 30 kg/m^2^. The following variables were included in this study as health determinants: current smoking status (yes/no), heavy alcohol consumption (yes/no), walking (yes/no), and muscle training (yes/no). We defined heavy alcohol consumption as at least seven or five cups of alcoholic drinks twice or more per week for men and women, respectively. Access to healthcare was evaluated with variables such as having undergone national health screening within the last 2 years (yes/no), having undergone cancer screening within the last 2 years (yes/no), and having visited an outpatient clinic within the last 2 weeks (yes/no). Finally, laboratory test results, such as those pertaining to total cholesterol (TC) levels (mg/dL), diastolic blood pressure (mmHg), FPG (mg/dL), and triglyceride concentration (TG) (mg/dL), were included in the analysis.

### Statistical Analysis

The complex sample analysis method was used to account for the multisampling method and different sample weights. Sample weight values assigned to each person were recalculated based on the primary sampling units as the data comprise those from the KNHANES 2010–2017. We applied sample weights to all calculation processes to reflect the demographic characteristics of the regions from where the participants were selected. The R package “survey” allows for the calculation of weighted percentages and means and the performance of regression analyses based on the unit of stratification and the cluster group.

The impact of risk factors on people with UDM was explored in comparison to that on those with/without DM. First, the weighted prevalence of each group was calculated. Variables based on qualitative and quantitative measurements are presented as weighted percentages with standard error (SE) and weighted means with SE, respectively. A chi-square test and analysis of variance (ANOVA), which were calculated with sampling weights, were used to evaluate the association between DM status and covariates. The multinomial logistic regression model was then employed to compare the impacts of the risk factors on the DM and UDM groups, using the without-DM group as the reference. The crude and age- and sex-adjusted models were constructed with sample weights for each variable. The results of the application of age- and sex-adjusted models for each variable are presented in forest plots to evaluate the magnitude of impact of each variable on DM and UDM status. Finally, the logistic regression model with sample weights was used to confirm the difference in the selected variables between the DM and UDM groups. Univariate and multivariable analyses, adjusted for age and sex, were performed for each regression analysis. Additionally, multivariable logistic regression analysis was performed to compare the DM and UDM groups in terms of risk factors. The regression models in those analyses included variables that were significantly associated with DM status. The results are presented in the Supplementary Tables. The level of significance was set to *p* < 0.05. All analyses were performed using R software 3.6.1 (R Foundation for Statistical Computing, Vienna, Austria).

## 3. Results

Among the 64,759 people who participated in the KNHANES 2010–2017, 32,611 individuals remained after the application of the exclusion criteria (Figure 1). The estimated age- and sex-adjusted prevalence rates of DM and UDM in Korea increased from 2010 to 2017 (DM: odds ratio (OR): 1.03; 95% confidence interval (CI), 1.01, 1.05; UDM: OR: 1.06; 95% CI: 1.02, 1.09) (Figure 1). The average age of the DM group (61.55 ± 0.26) was higher than that of the other groups (without DM, 42.62 ± 0.15; UDM, 53.21 ± 0.44). The proportion of women in the without-DM group was higher (54% ± 0.34) than that of men (46% ± 0.34), while the proportion of men in the DM and UDM groups (53.14 ± 1.02%; 63.57 ± 1.56%, respectively) was higher (Table 1). The waist circumference (89.49 ± 0.34 cm), BMI (26.26 ± 0.14 kg/m^2^), TC (206.06 ± 1.62 mg/dL), systolic blood pressure (126.82 ± 0.58 mmHg), diastolic blood pressure (80.88 ± 0.40 mmHg), FPG (155.99 ± 1.67 mg/dL), and TG (220.93 ± 7.44 mg/dL) values were the highest in the UDM group (Table 1).

**Figure 2:**
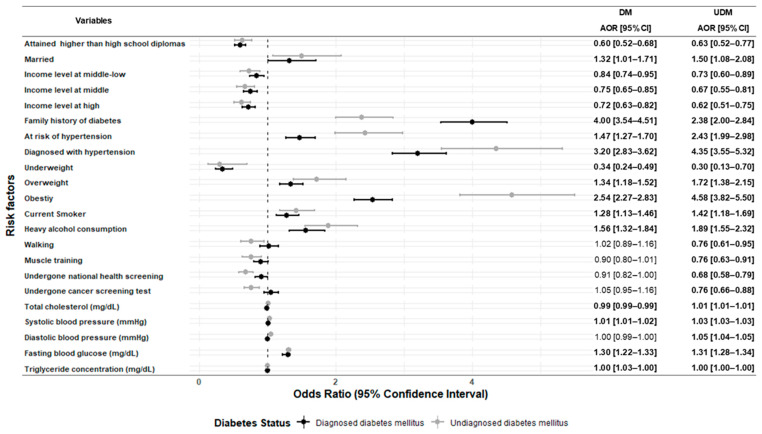
Forest plot of the association between baseline risk factors and DM/UDM status. The analysis was performed using an age- and sex-adjusted multinomial logistic regression model. The without-DM group was used as the reference. The ORs and 95% confidence intervals are drawn on the graph; dark-colored points with lines indicate DM and gray-colored points with lines indicate UDM. ORs with 95% CIs in bold style indicate a *p*-value of <0.05. DM, diabetes mellitus; UDM, undiagnosed diabetes mellitus; AOR, adjusted odds ratio.

Table 2 and Figure 2 show the differences in the ORs of each variable between the DM and UDM groups, using the without-DM group as the reference. As seen in Table 2, the distributions of all risk factors, except for muscle training and undergoing national health and cancer screening, were significantly different between the without-DM and UDM groups. The distributions of overweightness and obesity were significantly different between the DM and UDM groups (OR: 2.12; 95% CI: 1.70, 2.65; OR: 5.20; 95% CI: 4.34, 6.24, respectively); the magnitude of the ORs was larger than that of those observed in the DM group (OR: 1.67; 95% CI: 1.49, 1.88; OR: 2.73; 95% CI: 2.47, 3.02, respectively). The ORs of heavy alcohol consumption in the UDM group were significantly higher than the corresponding values in the without-DM group (OR: 1.82; 95% CI: 1.51, 2.20), while this risk factor was nonsignificantly associated with DM status.

In Figure 2, the distributions of risk factors in the DM and UDM groups were investigated using an age- and sex-adjusted multinomial regression model. The adjusted odds ratios (AORs) of family history of diabetes in the DM and UDM groups (AOR: 4.00; 95% CI: 3.54, 4.51; AOR: 2.38; 95% CI: 2.00, 2.84) were higher than the corresponding AOR of the without-DM group. The prevalence rates of hypertension (AOR: 3.20; 95% CI: 2.83, 3.62; AOR: 4.35; 95% CI: 3.55, 5.32, respectively) and obesity (AOR: 5.33; 95% CI: 4.41, 6.43; AOR: 10.87; 95% CI: 8.70, 13.57, respectively) were higher in the DM and UDM groups. For health determinant variables, the DM and UDM groups showed higher AORs in heavy alcohol consumption (AOR: 1.56; 95% CI: 1.32, 1.84; AOR: 1.89; 95% CI: 1.55, 2.32, respectively). Those in the UDM group were less likely to walk for 30 min/day for at least 5 days per week or to perform muscle training at least 2 days per week (AOR: 0.76; 95% CI: 0.61, 0.95; AOR: 0.76; 95% CI: 0.63, 0.91, respectively); the differences between the with- and without-DM groups were not significant (AOR: 1.02, 95% CI: 0.89, 1.16; AOR: 0.90; 95% CI: 0.80, 1.01, respectively). Concerning access to healthcare, the UDM group was less likely to have undergone national health and cancer screening tests (AOR: 0.68; 95% CI: 0.58, 0.79; AOR: 0.76; 95% CI: 0.66, 0.88, respectively).

Table 3 shows the characteristics of the UDM group, with the DM group used as the reference. The OR of a family history of diabetes was lower in the UDM group than in the DM group (AOR: 0.54; 95% CI: 0.3, 0.66). The ORs of hypertension and obesity were higher in the UDM group than in the DM group (AOR: 1.32; 95% CI: 1.06, 1.64 AOR: 1.80; 95% CI: 1.37, 2.36). The differences in health determinant variables, such as high alcohol intake, walking, and muscle training, were not significant between the DM and UDM groups. The OR of having undergone national health and cancer screening tests was lower in the UDM group than in the DM group (AOR: 0.74; 95% CI: 0.62, 0.89; AOR: 0.71; 95% CI: 0.60, 0.85, respectively).

## 4. Discussion

In this study, we explored the characteristics of individuals with UDM and compared them with those of individuals classified as with and without DM using the KNHANES 2010–2017 data. We assessed the prevalence of people with DM and UDM, as well as the association between risk factors and DM status. Our findings revealed the impact of various risk factors on the UDM group, comparing them to the with-/without-DM groups; this provided a better understanding of the characteristics of UDM. The results showed that individuals with UDM shared common baseline risk factors with those with DM. In addition, the distributions differed between individuals with UDM and DM in terms of risk factors such as health status, healthcare accessibility, and laboratory tests. Variables such as hypertension status, obesity, and undergoing national health and cancer screening tests were most strongly associated with the risk of UDM.

**Figure 3:**
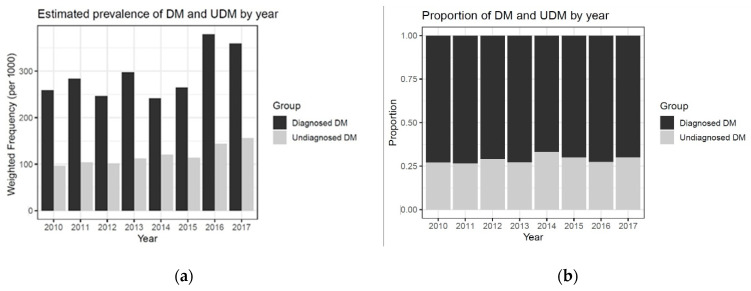
(**a**) Estimated prevalence of diagnosed and undiagnosed diabetes mellitus by year; (**b**) Proportions of patients with diagnosed and undiagnosed diabetes mellitus among all patients.

We also confirmed that the prevalence of UDM and DM increased since 2010 (Figure 3). Approximately 11% of our participants had DM, and approximately 29% of them were unaware of their prior DM status (Figure 3); this was less than the corresponding global and South-East Asia rate (47.9%) and slightly less than those reported by a previous U.S. survey (30% and 57%, respectively) [1,11,12]. Previous studies reported a significant upward trend in the prevalence of UDM and DM among Korean adults based on the KNHANES 2005–2015 data [4]. Our results showed that the estimated prevalence rates of DM and UDM increased from 2015 to 2017.

We observed the differences in the answers to the survey questions between the UDM and DM groups. The ORs of sex (male), hypertension, BMI, and obesity were higher in the UDM group than in the DM group. Furthermore, the ORs of age, family history of diabetes, health screening within the last 2 years, and cancer screening within the last 2 years were lower in the UDM group than in the DM group. Collectively, compared to the DM group, the patients in the UDM group were more likely to be young and male, with high BMI and blood pressure. Moreover, those in the UDM group were also more likely to believe that they were healthy but were less likely to have undergone cancer or national health screening tests or have a family history of DM.

In this study, the status of individuals with DM and UDM was more strongly associated with demographic characteristics compared to that of people without DM. Nevertheless, there was no significant difference between patients with DM and those with UDM. The demographic characteristics of patients with DM and UDM showed consistent results in previous studies [13,14,15,16]. Lower education attainment and income level have been linked as predictive factors to a high risk of diabetes [17,18]. Meanwhile, such characteristics in people with UDM were not significantly different from those in people with DM. Marital status was also significantly associated with DM and UDM status. Although previous studies have shown consistent results in terms of such an association [13,16], further studies are required to evaluate the link between marital status and DM.

Hypertension is an emerging major risk factor for type 2 DM. Reported works have shown that elevated blood pressure values are significantly associated with chronic inflammation and endothelial dysfunction [19,20], which are indicators of diabetes risk [19,21]. In addition, obesity, lipid profile, and blood pressure are risk factors for developing hypertension and type 2 DM [22]; thus, people with hypertension are more likely to develop type 2 DM. Previous studies have shown that people with hypertension are at greater risk of developing DM [23]. Analogously, our study showed significantly higher ORs of hypertension in the DM and UDM groups than in the without-DM group. Furthermore, the OR of having hypertension was significantly lower in the UDM group than in the DM group (Table 3), which was consistent with the results of previous studies [24,25,26]. However, the difference in the distribution of hypertension status between the DM and UDM groups was not significant in the multivariable logistic regression model when controlling for age, sex, family history of diabetes, obesity, and undergoing cancer screening tests (Supplementary Table S4). In addition, the direction of this association changed from negative to positive when age and sex were controlled for in the logistic regression model; the ORs for systolic and diastolic blood pressure were significantly higher in the UDM group than in the DM group. Our findings indicate that the UDM group may be at a higher risk of developing hypertension and complications related to DM. In addition, Lee et al. reported that people with UDM were more likely to be at risk of undiagnosed hypertension; in those with hypertension, it was more likely to remain uncontrolled [24].

Obesity and BMI are well-established indicators of DM. Our study confirmed that people with UDM were more likely to be obese and have higher BMI values than those classified as with or without DM. People with high BMI values are more likely to have high levels of non-esterified fatty acids, glycerol, hormones, cytokines, pro-inflammatory markers, and other substances, which induce the development of insulin resistance [27]. Consequently, the failure of β-islet cells of the pancreas could lead to a lack of blood glucose control, which eventually leads to the development of type 2 DM [27]. Previous studies have also shown that the prevalence of obesity among people with DM and UDM is higher than that among people without DM [24,28].

The results of several laboratory tests, such as those pertaining to TC and TG, showed greater abnormalities in the UDM group than in the with- and without-DM groups. The age- and sex-adjusted ORs of TC and TG were significantly higher in the UDM group than in the DM group. Previous studies have shown that the levels of such variables in the UDM group were significantly higher than those in the DM and without-DM groups in Korea [10,24,28]. High TC and TG levels have been reported to be positively associated with a person’s diabetes status; in particular, increases in the TG level over time were found to increase the risk of diabetes development [29]. Meanwhile, people with DM may have implemented lifestyle-related measures in addition to taking medications for dyslipidemia, which may have resulted in significantly higher average levels of TC and TG in the UDM group than in the DM group [30].

Regarding hypertension, obesity, and screening tests, our results showed higher values and averages in the UDM group than in the other groups. Although some UDM cases may be diagnosed in the early stages, people with UDM predominantly tend to prolong their status for several years, consequently aggravating the risk of developing DM-related complications. Furthermore, the higher risk of complications in the UDM group is evidenced by the results of the analysis that explored other variables, such as health-behavior-related indicators and access to healthcare.

Smoking status is a well-established indicator of diabetes development. As shown in previous studies, the odds of having a current smoking habit were significantly higher in the DM and UDM groups than in the group without DM [31]. However, the differences in the ORs of being a smoker between the DM and UDM groups were not significant after controlling for age and sex; the adjusted OR was higher in the UDM group than in the DM group when both were compared to the group without DM (adjusted OR in UDM: 1.416; adjusted OR in DM: 1.283). Although previous studies have shown a higher prevalence of a current smoking habit in the UDM group than in the DM group, they did not clarify whether this difference was significant [24,26].

The impact of alcohol consumption on DM varies depending on the amount and frequency of alcohol intake. Baliunas et al., who performed a meta-analysis that reviewed 20 cohort studies, found a U-shaped relationship between the alcohol consumption level and DM in men and women [32]. Interestingly, while moderate alcohol consumption may protect against DM [33], heavy drinking could increase the risk of DM development. Our study analyzed the odds of heavy drinking across the groups. The definition of heavy drinking in this study followed that used by the KCDC. As shown in previous studies [10,24,33,34], the ORs of heavy drinking in the DM and UDM groups were higher than that in the without-DM group. As in the case of smoking status, the estimated adjusted ORs in the UDM group were more significant than those in the DM group, but the difference in the ORs between the UDM and DM groups was not significant.

The difference in the level of physical activity among the groups was investigated using variables such as walking for 30 min/day and performing muscle training exercises for at least 5 and 2 days per week, respectively. The differences in the levels of physical activity between the DM and UDM groups were not significant; however, the UDM group was significantly less likely to achieve the recommended level of physical activity than the group without DM. Anue et al. found an inverse association between physical activity and DM risk [35]. The inverse association between walking and DM risk was found to be significant [36,37,38]. In addition, walking for ≥30 min for 5 days a week was found to improve the degree of glycemic control [39]. Participation in high-intensity physical activity provided robust protection against UDM risk [35]. Furthermore, Lee et al. reported a significant inverse association between regular muscle training and FPG, fasting insulin, and homeostasis model assessment of insulin resistance values [40].

This study elaborated the potential impact of undergoing a screening test to examine UDM status. Diabetes is an ambulatory care-sensitive condition, which means that early detection and provision of primary care, such as that aimed at glucose control, are critical to reducing the risk of diabetes-related illness and complications [41]. According to a report from the Organization for Economic Co-operation and Development (OECD) in 2015, the number of doctor consultations per person in Korea was the highest among all OECD countries [42]. In addition, Kim and Cheng found that a higher number of doctor consultations significantly reduced the rate of avoidable diabetes-related hospitalizations in Korea [43]. However, our study showed that patients with UDM were less likely to access healthcare services, such as national health and cancer screening, compared to those classified as with and without DM. Besides this, the degree of impairment in the FPG levels was higher in the UDM group than in the DM group, implying that people with UDM have poor glucose control and do not receive treatment. Therefore, a high frequency of hospital visits does not guarantee early detection of DM, unless a person undergoes screening tests. In Korea, efforts toward reducing the prevalence of UDM are insufficient, because the proportion of UDM cases among people with DM has not changed over time (Figure 3).

Patients with UDM should be encouraged to participate in the screening test for detecting DM. Improvement of the participation rate for the diabetes-screening test may significantly reduce the burden of DM. Studies have provided recommendations for screening tests for specific populations at risk of UDM [24,25,44,45]. The present study confirmed that DM and UDM share baseline risk factors, and the health status, including hypertension status, obesity, and BMI, in people with UDM was worse than that in people with DM. Several studies that have been performed in various countries, such as the United States, Germany, Japan, China, and Ethiopia, showed similar characteristics of patients with UDM to those reported in our study [15,25,26,29,44,45]. The authors of these studies also suggested active encouragement to conduct screening tests for individuals at risk of UDM. Therefore, it is necessary to implement public health campaigns or programs to improve screening test participation in vulnerable populations. Previous studies have also developed simple screening methods for DM for specific populations using risk score models [10,46,47,48]. We suggest that healthcare providers use the existing screening methods for DM with people at risk of UDM along with public health actions.

Our study had several limitations. First, it had a cross-sectional design. Therefore, the results of the analysis did not reveal real casual relationships. However, most of the observed associations were comparable to those of previous prospective cohort, case–control, and cross-sectional studies. Second, our study had conventional limitations associated with the use of a survey. The presence of recall bias could not be ruled out; thus, some participants may have been misclassified due to incorrect responses to our questions. Third, our study could not differentiate the type of DM. However, a previous study showed that the proportion of type 1 DM among all types of diabetes was 6% and that the prevalence decreased during 2011–2013 [49]. Thus, in this study, the impact of the included patients with type 1 DM on the overall results may be negligible. Fourth, we did not include the HbA1c level in the definition of DM, as the HbA1c level test was conducted only in people who were diagnosed with DM in KHNAES IV. Therefore, the numbers of people with DM and UDM may be underreported. Fifth, the present study may not rule out potential bias, as individuals who had missing data on diabetes mellitus status were excluded.

Despite these limitations, the present study had several strengths. Our study was based on a large population-based sample that represented the Korean population. To our knowledge, this KNHANES study was performed using the largest and latest dataset obtained over the last 5 years. In addition, our study newly found issues regarding access to healthcare services in people with UDM. Therefore, this finding supports our suggestion that a public health intervention targeting people at high risk of UDM in Korea is necessary.

## 5. Conclusions

In conclusion, our results indicate the urgent need for public health intervention to prevent UDM development in high-risk populations. The American Diabetes Association has established a list of recommendations for the prevention of DM in terms of lifestyle intervention, pharmacologic intervention, prevention of cardiovascular disease, and diabetes self-management education and support [50]. However, people with UDM may not want to follow these recommendations. The primary reason for this behavior could be a false belief regarding their health status. Although the differences in the health-status-related parameters, such as hypertension, BMI, obesity, and screening test results, between the DM and UDM groups in our study were not significant, patients with UDM were more likely to answer “good” or “very good” to the question on self-reported health status, and they reported a similar quality of life to individuals without DM. Besides this, the OR of family history of DM, which is the major risk factor of DM and considered an important tool for screening UDM [51], was higher in the UDM group than in the without-DM group but lower than in the DM group. Thus, people with UDM tend to be more likely to be careless in their behaviors, which increases their exposure to DM risk factors. Interventions with concrete implementation strategies are necessary to encourage people at high risk of UDM to prevent the development of DM, thereby reducing the burden of DM and DM-related diseases. Therefore, future studies should focus on finding specific groups at a high risk of UDM to detect such patients more efficiently. Although this study explored the overall impact of baseline risk factors on people with UDM, additional potential confounders for the association between each variable and DM status were not investigated when constructing the regression models. Instead, we estimated the impact of the risk factors on DM status, controlling for age and sex. Thus, several risk factors, such as marital status and healthcare accessibility, should be analyzed in depth regarding UDM incidence.

## Figures and Tables

**Table 1:** Basic characteristics of the without-DM, with-DM, and UDM groups.

Variables	Basic Characteristics									
	Without DM			DM			UDM			*p*-Value
	*n*	%(mean)	SD	*n*	%(mean)	SD	*n*	%(mean)	SD	
Demographic characteristics										
Age	27,809	42.62	0.15	3626	61.55	0.26	1176	53.21	0.44	<0.0001 ^b^
20–29	4299	23.52	0.42	14	0.86	0.26	29	4.21	0.85	<0.0001 ^a^
30–39	6151	23.57	0.42	73	2.98	0.39	99	10.54	1.17	
40–49	5546	21.69	0.36	263	11.57	0.76	231	26.16	1.66	
50–59	5038	16.62	0.28	720	26.66	0.95	295	28.72	1.63	
60–69	3694	8.29	0.19	1230	29.13	0.88	295	17.68	1.11	
≥70	3081	6.31	0.18	1326	28.79	0.86	227	12.69	0.94	
Sex										
Female	17,129	54.00	0.34	1828	46.86	1.02	500	36.43	1.56	<0.0001 ^a^
Male	10,680	46.00	0.34	1798	53.14	1.02	676	63.57	1.56	
Educational attainment										
High school or less	14,508	45.82	0.52	2982	79.48	0.88	842	67.01	1.77	<0.0001 ^a^
Higher than high school	12,901	52.62	0.52	568	18.22	0.84	292	29.61	1.73	
No response	400	1.55	0.13	76	2.29	0.30	42	3.38	0.64	
Marital status										
Not married	5219	27.24	0.45	90	4.00	0.46	72	9.74	1.24	<0.0001 ^a^
Married	22,586	72.76	0.45	3535	95.97	0.46	1103	90.24	1.24	
No response	4	0.00	0.00	1	0.03	0.03	1	0.02	0.02	
Income level										
Low	6435	24.51	0.45	995	29.01	0.96	358	31.86	1.63	<0.0001 ^a^
Middle–low	6955	25.13	0.42	918	25.14	0.87	288	23.96	1.49	
Middle	7139	25.26	0.40	832	22.48	0.84	266	20.95	1.35	
High	7109	24.39	0.51	857	22.70	0.89	247	21.82	1.46	
No response	171	0.71	0.08	24	0.67	0.17	17	1.42	0.40	
Health status										
Family history of diabetes										
No	23,621	84.11	0.28	2834	74.24	0.95	927	75.28	1.60	<0.0001 ^a^
Yes	4188	15.89	0.28	792	25.76	0.95	249	24.72	1.60	
Hypertension status										
Normal	14,963	57.58	0.42	619	19.71	0.84	225	20.72	1.41	<0.0001 ^a^
At high risk	6615	24.22	0.34	650	20.02	0.86	321	28.78	1.58	
Hypertension	6175	18.20	0.29	2347	60.27	1.01	627	50.50	1.79	
Obesity										
Underweight	1487	5.69	0.18	46	1.11	0.19	10	0.60	0.26	<0.0001 ^a^
Normal	12,671	45.18	0.37	1027	28.68	0.94	237	19.31	1.38	
Overweight	6343	22.33	0.30	897	23.71	0.85	250	20.25	1.41	
Obesity	7255	26.60	0.33	1648	46.25	1.00	678	59.59	1.73	
No response	53	0.20	0.03	8	0.25	0.10	1	0.25	0.25	
BMI (kg/m^2^)	27,756	23.21	0.03	3618	25.04	0.08	1175	26.26	0.14	<0.0001 ^b^
Health determinants										
Current smoking status										
No	22,581	76.82	0.36	2901	76.68	0.87	867	67.57	1.72	<0.0001 ^a^
Yes	5064	22.62	0.36	669	21.51	0.86	287	30.35	1.69	
No response	164	0.57	0.05	56	1.81	0.27	22	2.08	0.51	
Heavy alcohol consumption										
No	25,034	87.90	0.26	3272	87.04	0.78	969	79.18	1.46	<0.0001 ^a^
Yes	2608	11.54	0.26	304	11.30	0.72	188	19.09	1.43	
No response	167	0.56	0.05	50	1.66	0.27	19	1.73	0.47	
Walking										
No	9180	30.73	0.63	1055	28.76	1.02	332	28.91	1.65	<0.0001 ^a^
Yes	5561	19.77	0.45	686	17.60	0.84	174	14.03	1.23	
No response	13,068	49.50	0.91	1885	53.64	1.33	670	57.06	1.91	
Muscle training										
No	21,875	76.77	0.34	2904	79.12	0.84	917	77.51	1.49	<0.0001 ^a^
Yes	5528	21.64	0.33	644	18.52	0.81	216	18.67	1.38	
No response	406	1.58	0.13	78	2.36	0.31	43	3.82	0.71	
Healthcare accessibility										
Undergone national health screening										
No	10,479	41.29	0.41	1196	33.70	0.97	459	41.69	1.77	<0.0001 ^a^
Yes	16,972	57.30	0.41	2370	64.46	0.98	681	55.26	1.79	
No response	358	1.40	0.12	60	1.85	0.28	36	3.05	0.62	
Undergone cancer screening										
No	12,568	52.34	0.41	1328	38.06	1.00	524	49.41	1.70	<0.0001 ^a^
Yes	14,879	46.24	0.40	2237	60.07	1.01	614	47.42	1.68	
No response	362	1.42	0.12	61	1.87	0.28	38	3.17	0.63	
Laboratory tests										
Total cholesterol (mg/dL)	27,808	188.18	0.28	3625	175.70	0.79	1176	206.06	1.62	<0.0001 ^b^
Systolic blood pressure (mmHg)	27,776	114.60	0.13	3621	125.70	0.34	1175	126.82	0.58	<0.0001 ^b^
Diastolic blood pressure (mmHg)	27,776	74.89	0.09	3621	74.64	0.22	1175	80.88	0.40	<0.0001 ^b^
Fasting blood glucose (mg/dL)	27,809	89.43	0.05	3626	139.64	0.86	1176	155.99	1.67	<0.0001 ^b^
Triglyceride concentration (mg/dL)	27,808	120.34	0.77	3625	168.96	2.95	1176	220.93	7.44	<0.0001 ^b^

^a^ Chi-square test was conducted for categorical variables; ^b^ ANOVA was employed for evaluating continuously measured variables. BMI, body mass index; DM, diabetes mellitus; SD, standard deviation; UDM, undiagnosed diabetes mellitus.

**Table 2:** Results of the analysis investigating the crude association between baseline risk factors and DM/UDM status using multinomial logistic regression models. The without-DM group was used as the reference.

Variables	DM ^a,b^	UDM ^a,b^
	OR [95% CI]	OR [95% CI]
Demographic characteristics		
Age		
20–29	Reference	Reference
30–39	3.45 [1.80–6.61]	2.45 [1.53–3.92]
40–49	14.45 [7.73–26.98]	6.68 [4.30–10.38]
50–59	43.67 [23.70–80.44]	9.64 [6.29–14.77]
60–69	95.52 [51.77–176.24]	11.90 [7.80–18.16]
≥70	124.08 [67.45–228.24]	11.23 [7.23–17.44]
Sex		
Female	Reference	Reference
Male	1.34 [1.22–1.46]	2.05 [1.79–2.36]
Educational attainment		
High school or less	Reference	Reference
Higher than high school	0.20 [0.18–0.22]	0.39 [0.33–0.45]
Marital status		
Not married	Reference	Reference
Married	8.96 [7.07–11.35]	3.45 [2.61–4.55]
Income level		
Low	Reference	Reference
Middle–low	0.85 [0.76–0.95]	0.74 [0.61–0.89]
Middle	0.75 [0.67–0.85]	0.63 [0.53–0.77]
High	0.85 [0.76–0.95]	0.74 [0.61–0.89]
Health status		
Family history of diabetes		
No	Reference	Reference
Yes	1.84 [1.66–2.03]	1.71 [1.44–2.03]
Hypertension status		
Normal	Reference	Reference
At high risk	2.41 [2.10–2.78]	3.30 [2.71–4.03]
Hypertension	9.67 [8.67–10.80]	7.71 [6.43–9.25]
Obesity		
Underweight	0.31 [0.22–0.43]	0.25 [0.11–0.58]
Normal	Reference	Reference
Overweight	1.67 [1.49–1.88]	2.12 [1.70–2.65]
Obesity	2.73 [2.47–3.02]	5.20 [4.34–6.24]
Health determinants		
Current smoking status		
No	Reference	Reference
Yes	3.25 [2.27–4.66]	4.35 [2.61–7.25]
Heavy alcohol consumption		
No	Reference	Reference
Yes	0.99 [0.85–1.15]	1.82 [1.51–2.20]
Walking		
No	Reference	Reference
Yes	0.96 [0.84–1.09]	0.74 [0.60–0.93]
Muscle training		
No	Reference	Reference
Yes	0.83 [0.74–0.93]	0.84 [0.70–1.01]
Healthcare accessibility		
Undergone national health screening		
No	Reference	Reference
Yes	1.38 [1.26–1.51]	0.96 [0.83–1.11]
Undergone cancer screening		
No	Reference	Reference
Yes	1.78 [1.63–1.95]	1.08 [0.94–1.24]
Laboratory tests		
Total cholesterol (mg/dL)	0.99 [0.99–0.99]	1.02 [1.01–1.01]
Systolic blood pressure (mmHg)	1.00 [0.99–1.00]	1.05 [1.05–1.06]
Diastolic blood pressure (mmHg)	1.04 [1.04–1.04]	1.04 [1.04–1.05]
Fasting blood glucose (mg/dL)	1.34 [1.31–1.37]	1.35 [1.32–1.38]
Triglyceride concentration (mg/dL)	1.00 [1.00–1.00]	1.01 [1.00–1.01]

^a^ Multinomial logistic regression model with the without-DM group as a reference was used for the analysis; ^b^ ORs with 95% CIs in bold style indicate a *p*-value of <0.05. CI, confidence interval; DM, diabetes mellitus; OR, odds ratio; UDM, undiagnosed diabetes mellitus.

**Table 3:** Results of the analysis investigating the association between baseline risk factors and UDM status using logistic regression models. The DM group was used as the reference.

Variables	Crude Model ^a,c^	Age-/Sex-Adjusted Model ^b,c^
	OR [95% CI]	AOR [95% CI]
Demographic characteristics		
Age		
20–29	Reference	
30–39	0.71 [ 0.32–1.58]	
40–49	0.46 [0.22–0.99]	
50–59	0.22 [0.11–0.46]	
60–69	0.13 [0.06–0.26]	
≥70	0.09 [0.04–0.19]	
Sex		
Female	Reference	
Male	1.54 [1.31–1.80]	
Educational attainment		
High school or less	Reference	Reference
Higher than high school	1.94 [1.60–2.36]	1.11 [0.89–1.39]
Marital status		
Not married	Reference	Reference
Married	0.49 [0.27–0.55]	1.05 [0.71–1.56]
Income level		
Low	Reference	Reference
Middle–low	0.88 [0.70–1.10]	0.89 [0.71–1.11]
Middle	0.84 [0.68–1.04]	0.90 [0.72–1.13]
High	0.878 [0.70–1.10]	0.94 [0.74–1.18]
Health status		
Family history of diabetes		
No	Reference	Reference
Yes	0.93 [0.77–1.13]	0.54 [0.43–0.66]
Hypertension status		
Normal	Reference	Reference
At high risk	1.37 [1.09–1.72]	1.65 [1.29–2.11]
Hypertension	0.80 [0.65–0.98]	1.32 [1.06–1.64]
Obesity		
Underweight	0.81 [0.33–2.02]	0.98 [0.36–2.65]
Normal	Reference	Reference
Overweight	1.27 [1.00–1.62]	1.22 [0.95–1.58]
Obesity	1.90 [1.55–2.34]	1.67 [1.34–2.08]
Health determinants		
Current smoking status		
No	Reference	Reference
Yes	1.62 [1.34–1.95]	1.06 [0.86–1.31]
Heavy alcohol consumption		
No	Reference	Reference
Yes	1.84 [1.46–2.32]	1.20 [0.93–1.55]
Walking		
No	Reference	Reference
Yes	0.78 [0.61–1.00]	0.78 [0.60–1.02]
Muscle training		
No	Reference	Reference
Yes	1.01 [0.83–1.24]	0.85 [0.68–1.05]
Healthcare accessibility		
Undergone national health screening		
No	Reference	Reference
Yes	0.70 [0.59–0.83]	0.74 [0.62–0.89]
Undergone cancer screening		
No	Reference	Reference
Yes	0.61 [0.52–0.71]	0.71 [0.60–0.85]
Laboratory tests		
Total cholesterol (mg/dL)	1.02 [1.02–1.02]	1.02 [1.02–1.02]
Systolic blood pressure (mmHg)	1.00 [1.00–1.01]	1.01 [1.01–1.02]
Diastolic blood pressure (mmHg)	1.06 [1.05–1.07]	1.04 [1.03–1.05]
Fasting blood glucose (mg/dL)	1.01 [1.01–1.01]	1.01 [1.00–1.01]
Triglyceride concentration (mg/dL)	1.00 [1.00–1.00]	1.00 [1.00–1.00]

^a^ A logistic regression model was used for the analysis. The values of the DM group were used as references; ^b^ The association between each risk factor and UDM was analyzed using the age- and sex-adjusted logistic regression model; ^c^ ORs with 95% CIs in bold style indicate a *p*-value of <0.05. DM, diabetes mellitus; CI, confidence interval; OR, odds ratio; AOR, adjusted odds ratio; UDM, undiagnosed diabetes mellitus.

## Data Availability

The authors have no authority over the data, and the data is provided upon request to the Ministry of Health and Welfare.

## Supplementary Materials

The following are available online at https://www.mdpi.com/1660-4601/18/3/1195/s1, Table S1: Age subgroup analysis results for the impact of baseline risk factors on people with DM and UDM using an age- and sex-adjusted multinomial logistic regression model with the without-DM group as a reference, Table S2: Sex subgroup analysis results for the impact of baseline risk factors on people with DM and UDM using an age-adjusted multinomial logistic regression model with the without-DM group as a reference, Table S3: Age and sex subgroup analysis results for the impact of baseline risk factors on people with UDM using a multinomial logistic regression model with the with-DM group as a reference, Table S4: Results of the multivariable analysis investigating the association between the baseline risk factors and UDM status using a logistic regression model. The DM group was used as the reference.
