# Supplementary material for: Risk Factors of Undiagnosed Diabetes Mellitus among Korean Adults: A National Cross-Sectional Study Using the KNHANES Data
Source: Int J Environ Res Public Health. 2021 Jan 29;18(3):1195. doi: 10.3390/ijerph18031195 (PMC7908078; doi:10.3390/ijerph18031195)
Supplement: Supplementary file 1 [file ijerph-18-01195-s001.pdf]

Supplement table 1. Age subgroup analysis results for the impact of baseline risk factors on people with DM and UDM using age- and sex-adjusted multinomial logistic regression model with the without DM group as reference

Variables	Age <40		Age ≥40	
	Known	Unknown	Known	Unknown
	OR [95% CI]	OR [95% CI]	OR [95% CI]	OR [95% CI]
Demographic characteristics				
**Educational attainment**				
High school or less	*Reference*	*Reference*	*Reference*	*Reference*
Higher than high school	**0.30 [0.18-0.49]**	**0.63 [0.41-0.97]**	**0.69 [0.61-0.79]**	**0.70 [0.57-0.85]**
**Marital status**				
Not married	*Reference*	*Reference*	*Reference*	*Reference*
Married	1.05 [0.50-2.20]	0.82 [0.49-1.36]	**0.59 [0.44-0.81]**	**0.58 [0.38-0.90]**
**Employment status**				
No	*Reference*	*Reference*	*Reference*	*Reference*
Yes	0.91 [0.52-1.61]	1.05 [0.65-1.69]	**0.76 [0.68-0.84]**	**0.84 [0.70-1.00]**
**Income level**				
Low	*Reference*	*Reference*	*Reference*	*Reference*
Middle-low	0.66 [0.36-1.22]	0.75 [0.43-1.30]	**0.85 [0.75-0.96]**	**0.73 [0.59-0.89]**
Middle	0.80 [0.42-1.50]	0.61 [0.33-1.16]	**0.72 [0.63-0.82]**	**0.62 [0.51-0.76]**
High	0.55 [0.26-1.18]	1.22 [0.73-2.04]	**0.75 [0.66-0.86]**	**0.60 [0.48-0.74]**
Health status				
**Family history of diabetes**				
No	*Reference*	*Reference*	*Reference*	*Reference*
Yes	**4.80 [3.00-7.67]**	**3.19 [2.11-4.83]**	**3.66 [3.23-4.14]**	**1.92 [1.57-2.33]**
**Hypertension status**				
Normal	*Reference*	*Reference*	*Reference*	*Reference*

At high risk	1.53 [0.79-2.96]	**2.46 [1.44-4.20]**	**1.43 [1.23-1.66]**	**2.40 [1.93-2.99]**
Hypertension	**4.00 [2.06-7.75]**	**5.16 [2.83-9.39]**	**3.10 [2.74-3.51]**	**4.27 [3.47-5.25]**
**Obesity**				
Underweight	0.38 [0.05-2.79]	**0.12 [0.02-0.96]**	**0.37 [0.26-0.53]**	**0.38 [0.16-0.93]**
Normal	*Reference*	*Reference*	*Reference*	*Reference*
Overweight	1.38 [0.60-3.21]	**2.35 [1.06-5.24]**	**1.30 [1.15-1.47]**	**1.59 [1.27-2.01]**
Obesity	**3.21 [1.56-6.62]**	**9.53 [4.78-18.97]**	**2.42 [2.17-2.70]**	**4.00 [3.30-4.85]**
**BMI (kg/m2)**	**1.19 [1.12-1.26]**	**1.32 [1.26-1.38]**	**1.18 [1.16-1.20]**	**1.24 [1.22-1.27]**
**Waist circumference (cm)**	**1.07 [1.04-1.10]**	**1.12 [1.10-1.14]**	**1.08 [1.07-1.08]**	**1.09 [1.08-1.10]**
**Self-reported health status**				
Very bad	**17.92 [6.81-47.19]**	1.77 [0.24-13.09]	**2.87 [2.39-3.44]**	1.15 [0.77-1.74]
Bad	**4.48 [2.55-7.86]**	**1.74 [1.01-2.99]**	**1.98 [1.76-2.23]**	1.18 [0.96-1.46]
Normal				
Good	**0.32 [0.15-0.71]**	**0.54 [0.31-0.93]**	**0.48 [0.42-0.55]**	**0.60 [0.49-0.73]**
Very good	0.48 [0.11-2.07]	0.15 [0.02-1.07]	**0.36 [0.26-0.49]**	0.76 [0.53-1.09]
**EuroQol-5D (EQ-5D) index score**	**0.01 [0.00-0.03]**	0.52 [0.02-12.86]	**0.29 [0.21-0.40]**	0.57 [0.29-1.12]
**Health determinant**				
**Current smoking status**				
No	*Reference*	*Reference*	*Reference*	*Reference*
Yes	**1.84 [1.05-3.22]**	1.49 [0.91-2.44]	**1.23 [1.08-1.40]**	**1.33 [1.10-1.61]**
**Heavy alcohol consumption**				
No	*Reference*	*Reference*	*Reference*	*Reference*
Yes	1.68 [0.90-3.13]	1.46 [0.88-2.44]	**1.49 [1.25-1.77]**	**1.85 [1.48-2.31]**
**Walking**				
No	*Reference*	*Reference*	*Reference*	*Reference*
Yes	1.13 [0.58-2.22]	0.80 [0.43-1.49]	1.03 [0.91-1.17]	0.80 [0.63-1.01]

**Muscle training**				
No	*Reference*	*Reference*	*Reference*	*Reference*
Yes	0.74 [0.40-1.37]	0.66 [0.36-1.19]	0.89 [0.79-1.01]	**0.79 [0.65-0.96]**
Healthcare accessibility				
**Undergone the national health screening**				
No	*Reference*	*Reference*	*Reference*	*Reference*
Yes	0.65 [0.39-1.07]	**0.61 [0.40-0.94]**	**0.84 [0.76-0.92]**	**0.59 [0.50-0.69]**
**Undergone cancer screening test**				
No	*Reference*	*Reference*	*Reference*	*Reference*
Yes	0.62 [0.36-1.08]	0.67 [0.40-1.12]	0.95 [0.86-1.04]	**0.63 [0.54-0.73]**
**Outpatient visit**				
No	*Reference*	*Reference*	*Reference*	*Reference*
Yes	**3.82 [1.08-13.52]**	1.17 [0.48-2.84]	**1.51 [1.26-1.81]**	1.06 [0.79-1.41]
Laboratory test				
**Total cholesterol (mg/dL)**	1.00 [0.99-1.01]	**1.02 [1.01-1.02]**	**0.99 [0.98-0.99]**	**1.01 [1.01-1.01]**
**High-density lipoprotein (mg/dL)**	**0.97 [0.95-1.00]**	**0.94 [0.92-0.96]**	**0.96 [0.96-0.97]**	**0.97 [0.96-0.98]**
**Blood creatinine (mg/dL)**	0.10 [0.00-2.07]	**0.02 [0.00-0.20]**	**1.45 [1.04-2.01]**	**0.47 [0.28-0.78]**
**Estimated glomerular filtration rate (e-GFR) test**	**1.01 [1.00-1.02]**	**1.02 [1.01-1.03]**	**0.99 [0.99-1.00]**	**1.01 [1.00-1.01]**
**Blood urea nitrogen (mg/dL)**	**1.05 [1.00-1.10]**	1.03 [0.97-1.09]	**1.03 [1.02-1.05]**	**1.01 [1.00-1.03]**
**Systolic blood pressure (mmHg)**	**1.03 [1.01-1.05]**	**1.05 [1.04-1.07]**	**1.01 [1.01-1.01]**	**1.03 [1.02-1.03]**
**Diastolic blood pressure (mmHg)**	**1.04 [1.01-1.06]**	**1.05 [1.04-1.07]**	**0.99 [0.99-1.00]**	**1.04 [1.03-1.05]**
**Fasting blood glucose (mg/dL)**	**1.38 [1.28-1.48]**	**1.39 [1.29-1.49]**	**1.29 [1.26-1.32]**	**1.30 [1.27-1.33]**
**Triglyceride concentration (mg/dL)**	**1.00 [1.00-1.00]**	**1.00 [1.00-1.00]**	**1.00 [1.00-1.00]**	**1.00 [1.00-1.01]**

OR with 95% CI written in bold style indicates that p-value is less than 0.05

Supplement table 2. Sex subgroup analysis results for the impact of baseline risk factors on people with DM and UDM using age-adjusted multinomial logistic regression model with the without DM group as reference

**Variables**	**Male**		**Female**	
	**Known**	**Unknown**	**Known**	**Unknown**
	**OR [95% CI]**	**OR [95% CI]**	**OR [95% CI]**	**OR [95% CI]**
Demographic characteristics				
**Educational attainment**				
High school or less	*Reference*	*Reference*	*Reference*	*Reference*
Higher than high school	**0.67 [0.57-0.79]**	**0.74 [0.58-0.93]**	**0.45 [0.36-0.58]**	**0.43 [0.31-0.59]**
**Marital status**				
Not married	*Reference*	*Reference*	*Reference*	*Reference*
Married	**0.98 [0.98-0.98]**	**1.40 [1.40-1.40]**	**1.88 [1.13-3.12]**	**1.87 [1.06-3.29]**
**Employment status**				
No	*Reference*	*Reference*	*Reference*	*Reference*
Yes	1.01 [0.87-1.18]	**1.36 [1.08-1.72]**	**0.77 [0.67-0.88]**	0.87 [0.69-1.09]
**Income level**				
Low	*Reference*	*Reference*	*Reference*	*Reference*
Middle-low	**0.83 [0.70-1.00]**	0.82 [0.63-1.05]	**0.84 [0.70-1.00]**	**0.61 [0.46-0.80]**
Middle	**0.71 [0.59-0.87]**	**0.64 [0.49-0.83]**	**0.73 [0.62-0.87]**	**0.58 [0.44-0.78]**
High	0.83 [0.68-1.01]	**0.76 [0.59-0.99]**	**0.66 [0.55-0.80]**	**0.54 [0.40-0.72]**
Health status				
**Family history of diabetes**				
No	*Reference*	*Reference*	*Reference*	*Reference*
Yes	**4.06 [3.42-4.82]**	**2.41 [1.91-3.05]**	**4.00 [3.37-4.74]**	**2.35 [1.84-3.01]**
**Hypertension status**				
Normal	*Reference*	*Reference*	*Reference*	*Reference*

At high risk	1.21 [0.99-1.48]	**2.19 [1.67-2.89]**	**1.88 [1.51-2.34]**	**3.11 [2.25-4.29]**
Hypertension	**2.61 [2.21-3.08]**	**4.34 [3.29-5.73]**	**4.15 [3.43-5.03]**	**4.57 [3.32-6.28]**
**Obesity**				
Underweight	**0.23 [0.14-0.39]**	**0.31 [0.11-0.90]**	**0.51 [0.31-0.83]**	**0.24 [0.07-0.82]**
Normal	*Reference*	*Reference*	*Reference*	*Reference*
Overweight	**1.27 [1.06-1.52]**	**1.43 [1.08-1.89]**	**1.39 [1.16-1.65]**	**2.52 [1.73-3.69]**
Obesity	**2.14 [1.81-2.53]**	**3.31 [2.60-4.20]**	**2.97 [2.57-3.44]**	**8.54 [6.26-11.66]**
**BMI (kg/m2)**	**1.16 [1.14-1.19]**	**1.22 [1.19-1.26]**	**1.20 [1.18-1.22]**	**1.30 [1.26-1.33]**
**Waist circumference (cm)**	**1.07 [1.06-1.08]**	**1.08 [1.07-1.09]**	**1.08 [1.07-1.09]**	**1.12 [1.11-1.13]**
**Self-reported health status**				
Very bad	**3.11 [2.22-4.35]**	0.92 [0.45-1.89]	**2.71 [2.17-3.39]**	**1.21 [0.75-1.94]**
Bad	**2.25 [1.87-2.70]**	**1.38 [1.05-1.81]**	**1.89 [1.62-2.20]**	1.02 [0.77-1.37]
Normal				
Good	**0.43 [0.36-0.52]**	**0.52 [0.41-0.67]**	**0.53 [0.43-0.65]**	**0.70 [0.52-0.94]**
Very good	**0.31 [0.22-0.44]**	**0.56 [0.35-0.88]**	**0.45 [0.24-0.82]**	0.81 [0.48-1.34]
**EuroQol-5D (EQ-5D) index score**	**0.27 [0.15-0.49]**	0.63 [0.22-1.79]	**0.40 [0.26-0.62]**	1.31 [0.56-3.09]
**Health determinant**				
**Current smoking status**				
No	*Reference*	*Reference*	*Reference*	*Reference*
Yes	**1.26 [1.10-1.45]**	**1.41 [1.16-1.72]**	1.25 [0.92-1.69]	1.47 [0.94-2.30]
**Heavy alcohol consumption**				
No	*Reference*	*Reference*	*Reference*	*Reference*
Yes	**1.60 [1.33-1.91]**	**1.98 [1.60-2.46]**	1.17 [0.73-1.89]	1.42 [0.82-2.45]
**Walking**				
No	*Reference*	*Reference*	*Reference*	*Reference*
Yes	0.98 [0.81-1.20]	**0.66 [0.49-0.90]**	1.08 [0.89-1.33]	0.96 [0.70-1.31]

**Muscle training**				
No	*Reference*	*Reference*	*Reference*	*Reference*
Yes	0.99 [0.85-1.14]	**0.79 [0.63-0.98]**	**0.73 [0.59-0.91]**	**0.69 [0.48-0.98]**
Healthcare accessibility				
**Undergone the national health screening**				
No	*Reference*	*Reference*	*Reference*	*Reference*
Yes	0.95 [0.82-1.10]	**0.69 [0.56-0.84]**	0.88 [0.78-1.00]	**0.66 [0.53-0.83]**
**Undergone cancer screening test**				
No	*Reference*	*Reference*	*Reference*	*Reference*
Yes	**1.20 [1.04-1.38]**	**0.81 [0.67-0.99]**	0.97 [0.85-1.11]	**0.70 [0.56-0.87]**
**Outpatient visit**				
No	*Reference*	*Reference*	*Reference*	*Reference*
Yes	**1.68 [1.29-2.20]**	1.34 [0.93-1.93]	**1.41 [1.12-1.78]**	0.68 [0.43-1.06]
Laboratory test				
**Total cholesterol (mg/dL)**	**0.99 [0.99-0.99]**	**1.01 [1.01-1.01]**	**0.99 [0.98-0.99]**	**1.01 [1.01-1.02]**
**High-density lipoprotein (mg/dL)**	**0.97 [0.96-0.97]**	**0.97 [0.96-0.98]**	**0.96 [0.95-0.97]**	**0.95 [0.94-0.96]**
**Blood creatinine (mg/dL)**	**1.24 [1.03-1.50]**	**0.35 [0.19-0.67]**	**1.84 [1.36-2.50]**	**0.22 [0.09-0.54]**
**Estimated glomerular filtration rate (e-GFR) test**	1.00 [0.99-1.00]	**1.01 [1.00-1.01]**	**0.99 [0.99-1.00]**	**1.01 [1.01-1.02]**
**Blood urea nitrogen (mg/dL)**	**1.03 [1.01-1.05]**	1.01 [0.99-1.03]	**1.04 [1.03-1.06]**	1.02 [1.00-1.04]
**Systolic blood pressure (mmHg)**	**1.01 [1.01-1.02]**	**1.03 [1.03-1.04]**	**1.01 [1.01-1.02]**	**1.02 [1.02-1.03]**
**Diastolic blood pressure (mmHg)**	1.00 [0.99-1.01]	**1.05 [1.04-1.06]**	**0.99 [0.99-1.00]**	**1.04 [1.03-1.05]**
**Fasting blood glucose (mg/dL)**	**1.29 [1.25-1.32]**	**1.29 [1.26-1.33]**	**1.32 [1.28-1.36]**	**1.32 [1.29-1.36]**
**Triglyceride concentration (mg/dL)**	**1.00 [1.00-1.00]**	**1.00 [1.00-1.00]**	**1.01 [1.00-1.01]**	**1.01 [1.01-1.01]**

OR with 95% CI written in bold style indicates that p-value is less than 0.05

Supplement table 3. Age and sex subgroup analysis results for the impact of baseline risk factors on people with UDM using multinomial logistic regression model with the with DM group as reference

Variables	Age <40[a]	Age ≥40 [a]	Male[b]	Female[b]
	**OR [95% CI]**	**OR [95% CI]**	**OR [95% CI]**	**OR [95% CI]**
Demographic characteristics				
**Educational attainment**				
High school or less	*Reference*	*Reference*	*Reference*	*Reference*
Higher than high school	**2.10 [1.16-3.79]**	1.02 [0.80-1.29]	1.13 [0.86-1.47]	1.07 [0.73-1.57]
**Marital status**				
Not married	*Reference*	*Reference*	*Reference*	*Reference*
Married	0.79 [0.40-1.54]	1.03 [0.63-1.69]	1.07 [0.67-1.69]	0.97 [0.47-2.04]
**Employment status**				
No	*Reference*	*Reference*	*Reference*	*Reference*
Yes	1.11 [0.52-2.37]	1.09 [0.89-1.34]	1.15 [0.88-1.50]	1.08 [0.82-1.41]
**Income level**				
Low	*Reference*	*Reference*	*Reference*	*Reference*
Middle-low	1.13 [0.52-2.49]	0.87 [0.69-1.10]	0.99 [0.73-1.33]	0.76 [0.54-1.06]
Middle	0.75 [0.32-1.73]	0.93 [0.74-1.16]	0.90 [0.66-1.23]	0.91 [0.66-1.26]
High	2.17 [0.88-5.33]	0.85 [0.67-1.09]	0.91 [0.68-1.24]	0.99 [0.71-1.40]
Health status				
**Family history of diabetes**				
No	*Reference*	*Reference*	*Reference*	*Reference*
Yes	0.68 [0.39-1.16]	**0.51 [0.41-0.64]**	**0.54 [0.41-0.71]**	**0.53 [0.39-0.72]**
**Hypertension status**				

Normal	*Reference*	*Reference*	*Reference*	*Reference*
At high risk	1.61 [0.79-3.25]	**1.66 [1.28-2.15]**	**1.73 [1.24-2.41]**	**1.55 [1.07-2.24]**
Hypertension	1.27 [0.61-2.64]	1.34 [1.06-1.68]	**1.52 [1.13-2.04]**	1.06 [0.75-1.49]
**Obesity**				
Underweight	0.42 [0.02-7.76]	1.09 [0.43-2.75]	1.64 [0.52-5.17]	0.44 [0.10-2.03]
Normal	*Reference*	*Reference*	*Reference*	*Reference*
Overweight	1.77 [0.66-4.75]	1.18 [0.91-1.52]	1.13 [0.82-1.56]	1.45 [0.95-2.21]
Obesity	**3.11 [1.29-7.48]**	**1.57 [1.26-1.95]**	**1.46 [1.09-1.94]**	**2.13 [1.51-3.00]**
**BMI (kg/m2)**	**1.11 [1.04-1.18]**	**1.05 [1.02-1.07]**	**1.05 [1.01-1.08]**	**1.07 [1.03-1.11]**
**Waist circumference (cm)**	**1.05 [1.02-1.08]**	**1.01 [1.00-1.02]**	**1.01 [1.00-1.02]**	**1.02 [1.01-1.04]**
**Self-reported health status**				
Very bad	**0.10 [0.01-0.93]**	**0.38 [0.25-0.57]**	**0.29 [0.14-0.59]**	**0.41 [0.25-0.67]**
Bad	**0.38 [0.19-0.77]**	**0.55 [0.43-0.69]**	**0.57 [0.42-0.78]**	**0.46 [0.33-0.63]**
Normal				
Good	1.67 [0.65-4.28]	**1.45 [1.15-1.83]**	**1.41 [1.06-1.89]**	**1.57 [1.08-2.28]**
Very good	0.31 [0.02-3.76]	**2.50 [1.53-4.08]**	**2.68 [1.52-4.73]**	**1.71 [0.71-4.13]**
**EuroQol-5D (EQ-5D) index score**	**156.39 [2.06-11,887.51]**	**2.64 [1.20-5.84]**	2.16 [0.60-7.85]	**4.45 [1.78-11.17]**
Health determinant				
**Current smoking status**				
No	*Reference*	*Reference*	*Reference*	*Reference*
Yes	0.80 [0.41-1.56]	1.08 [0.87-1.34]	1.04 [0.83-1.31]	1.19 [0.71-2.00]
**Heavy alcohol consumption**				
No	*Reference*	*Reference*	*Reference*	*Reference*
Yes	0.83 [0.40-1.73]	1.25 [0.95-1.64]	1.19 [0.91-1.56]	1.28 [0.62-2.68]
**Walking**				
No	*Reference*	*Reference*	*Reference*	*Reference*

Yes	0.70 [0.29-1.67]	0.81 [0.62-1.05]	**0.68 [0.47-0.98]**	0.99 [0.68-1.42]
**Muscle training**				
No	*Reference*	*Reference*	*Reference*	*Reference*
Yes	0.91 [0.40-2.04]	0.86 [0.69-1.07]	0.84 [0.66-1.07]	0.88 [0.58-1.34]
Healthcare accessibility				
**Undergone the national health screening**				
No	*Reference*	*Reference*	*Reference*	*Reference*
Yes	0.96 [0.53-1.73]	**0.71 [0.59-0.86]**	**0.75 [0.59-0.96]**	**0.73 [0.56-0.94]**
**Undergone cancer screening test**				
No	*Reference*	*Reference*	*Reference*	*Reference*
Yes	1.08 [0.52-2.24]	**0.69 [0.58-0.82]**	**0.70 [0.56-0.88]**	**0.73 [0.56-0.94]**
**Outpatient visit**				
No	*Reference*	*Reference*	*Reference*	*Reference*
Yes	0.30 [0.08-1.15]	**0.67 [0.48-0.93]**	0.79 [0.53-1.19]	**0.45 [0.28-0.74]**
Laboratory test				
**Total cholesterol (mg/dL)**	**1.02 [1.01-1.03]**	**1.02 [1.01-1.02]**	**1.02 [1.01-1.02]**	**1.02 [1.01-1.02]**
**High-density lipoprotein (mg/dL)**	**0.96 [0.94-0.99]**	**1.01 [1.00-1.02]**	1.01 [1.00-1.02]	1.00 [0.99-1.01]
**Blood creatinine (mg/dL)**	0.40 [0.05-3.27]	**0.41 [0.28-0.60]**	**0.50 [0.32-0.79]**	**0.23 [0.11-0.46]**
**Estimated glomerular filtration rate (e-GFR) test**	1.01 [0.99-1.02]	**1.01 [1.00-1.01]**	**1.01 [1.00-1.01]**	**1.01 [1.00-1.01]**
**Blood urea nitrogen (mg/dL)**	0.98 [0.90-1.06]	**0.97 [0.96-0.99]**	**0.98 [0.96-1.00]**	**0.97 [0.95-0.99]**
**Systolic blood pressure (mmHg)**	**1.03 [1.00-1.06]**	**1.01 [1.01-1.02]**	**1.02 [1.01-1.03]**	**1.01 [1.00-1.02]**
**Diastolic blood pressure (mmHg)**	1.02 [0.99-1.05]	**1.04 [1.03-1.05]**	**1.04 [1.03-1.05]**	**1.04 [1.02-1.05]**
**Fasting blood glucose (mg/dL)**	**1.01 [1.00-1.01]**	**1.01 [1.00-1.01]**	**1.01 [1.00-1.01]**	**1.01 [1.00-1.01]**
**Triglyceride concentration (mg/dL)**	1.00 [1.00-1.01]	**1.00 [1.00-1.00]**	**1.00 [1.00-1.00]**	**1.00 [1.00-1.00]**

[a] Age- and sex-adjusted logistic regression model was used; [b] Age-adjusted logistic regression model was used; OR with 95% CI written in bold style indicates that p-value is less than 0.05

Supplementary Table 4. Results of the multivariable analysis investigating the association between the baseline risk factors and UDM status using a logistic regression model. The DM group was used as the reference.

Variables	Full model [a,c]	Reduced model [b,c]
	AOR [95% CI]	AOR [95% CI]
**Age**		
20-29	*Reference*	*Reference*
30-39	0.63 [0.26-1.55]	0.79 [0.34-1.83]
40-49	**0.41 [0.17-0.95]**	0.52 [0.24-1.16]
50-59	**0.23 [0.10-0.54]**	**0.24 [0.11-0.53]**
60-69	**0.16 [0.07-0.38]**	**0.13 [0.06-0.30]**
70≥	**0.14 [0.06-0.34]**	**0.09 [0.04-0.21]**
**Sex**		
Female	*Reference*	*Reference*
Male	**1.33 [1.08-1.63]**	**1.27 [1.07-1.51]**
**Educational attainment**		
High school or less	*Reference*	
Higher than high school	1.07 [0.84-1.38]	
**Marital status**		
Not married	*Reference*	
Married	1.17 [0.73-1.87]	
**Family history of diabetes**		
No	*Reference*	*Reference*
Yes	**0.51 [0.41-0.64]**	**0.53 [0.43-0.66]**
**Hypertension status**		
Normal	*Reference*	*Reference*
At high risk	0.95 [0.71-1.27]	**1.53 [1.18-1.97]**
Hypertension	**0.72 [0.54-0.97]**	1.10 [0.87-1.38]
**Obesity**		
Underweight	0.88 [0.40-1.96]	0.93 [0.38-2.27]
Normal	*Reference*	*Reference*
Overweight	1.17 [0.89-1.54]	1.23 [0.95-1.60]
Obese	**1.51 [1.19-1.91]**	**1.71 [1.36-2.15]**
**Current smoking status**		
No	*Reference*	
Yes	1.07 [0.85-1.35]	
**Heavy alcohol consumption**		
No	*Reference*	
Yes	1.07 [0.79-1.46]	
**Walking**		
No	*Reference*	
Yes	0.83 [0.63-1.1]	
**Undergone national health screening**		

No	*Reference*	
Yes	1.00 [0.75-1.33]	
**Undergone cancer screening test**		
No	*Reference*	*Reference*
Yes	0.81 [0.62-1.06]	**0.75 [0.63-0.89]**
**Total cholesterol (mg/dL)**	**1.02 [1.01-1.02]**	
**Systolic blood pressure (mmHg)**		
**Diastolic blood pressure (mmHg)**	**1.03 [1.02-1.05]**	
**Fasting blood glucose (mg/dL)**	**1.00 [1.00-1.01]**	
**Triglyceride concentration (mg/dL)**	1.00 [1.00-1.00]	

[a] A multivariable logistic regression model was used for the analysis. Variables that significantly explained the difference of ORs between UDM and DM on univariate analysis were included in the model; [b] Variables that significantly explained the difference in AORs between UDM and DM were retained from the full model. Model reduction was performed using backward stepwise elimination based on the magnitude of the p-value of each variable. Variables for laboratory test results were excluded at the initial step of reducing the model; [c] OR with 95% CIs in bold style indicate a p-value <.05

DM, diabetes mellitus; CI, confidence interval; OR, odds ratio; UDM, undefined diabetes mellitus
